# Hepatoprotective Effects and Antioxidant Properties of a Herbal Detoxifying Formula Against Chlorpyrifos-Induced Toxicity in Sprague-Dawley Rats

**DOI:** 10.3390/biology15010017

**Published:** 2025-12-21

**Authors:** Phraepakaporn Kunnaja, Sunee Chansakaow, Weerakit Taychaworaditsakul, Supaporn Intatham, Kanjana Jaijoy, Absorn Wittayapraparat, Pedcharada Yusuk, Ratchuporn Suksathan, Seewaboon Sireeratawong

**Affiliations:** 1 Department of Medical Technology, Faculty of Associated Medical Sciences, Chiang Mai University, Chiang Mai 50200, Thailand; phraepakaporn.k@cmu.ac.th; 2 Department of Pharmaceutical Sciences, Faculty of Pharmacy, Chiang Mai University, Chiang Mai 50200, Thailand; sunee.c@cmu.ac.th; 3 Department of Biochemistry, Faculty of Medicine, Chiang Mai University, Chiang Mai 50200, Thailand; weerakit.tay@cmu.ac.th; 4 Department of Pharmacology, Faculty of Medicine, Chiang Mai University, Chiang Mai 50200, Thailand; supaporn.inta@cmu.ac.th; 5 Clinical Research Center for Food and Herbal Product Trials and Development (CR-FAH), Faculty of Medicine, Chiang Mai University, Chiang Mai 50200, Thailand; 6 McCormick Faculty of Nursing, Payap University, Chiang Mai 50000, Thailand; kanjana_j@payap.ac.th; 7 Highland Research and Development Institute (Public Organization), Chiang Mai 50200, Thailand; absornw@hrdi.or.th (A.W.); npedcharada@hrdi.or.th (P.Y.); 8 Queen Sirikit Botanic Garden, The Botanical Garden Organization, Chiang Mai 50180, Thailand; r_spanuchat@yahoo.com

**Keywords:** hepatoprotection, detoxifying formula, chlorpyrifos, hepatic stellate cells, antioxidant, rosmarinic acid, apoptosis, *Thunbergia laurifolia*, *Embelia sessiliflora*

## Abstract

Chlorpyrifos (CPF), a widely used organophosphate pesticide, induces hepatotoxicity primarily through oxidative stress, acetylcholinesterase (AChE) inhibition, and inflammatory responses. This study evaluated the hepatoprotective potential of detoxifying formulations prepared from four medicinal plants, *Thunbergia laurifolia*, *Embelia sessiliflora*, *Morinda angustifolia*, and *Thunbergia coccinea*, in various ratios. Among these, a formulation composed of *T. laurifolia* and *E. sessiliflora* (1:1; Formula 04) showed the highest activity, with rosmarinic acid identified by compact mass spectrometry (CMS). Formula 04 demonstrated the greatest antioxidant and pro-apoptotic potential among the seven tested formulations, as confirmed by in vitro DPPH and superoxide radical assays and apoptosis assays in hepatic stellate LX-2 cells. In vivo, Sprague–Dawley rats exposed to CPF (16 mg/kg/day, oral gavage) received Formula 04 orally 30 min prior to CPF exposure in 6 cyclic dosing regimen for 18 days. The treatment restored AChE activity, increased superoxide dismutase levels, reduced glutathione levels, and decreased malondialdehyde, indicating attenuation of oxidative stress. Serum AST levels were significantly reduced compared with the CPF group, and histopathology revealed that the hepatic architecture was preserved. These findings demonstrate that Formula 04 exerts hepatoprotective effects against CPF-induced toxicity via redox regulation and cellular protection mechanisms, demonstrating its potential as a natural detoxifying agent.

## Simple Summary

Exposure to organophosphate pesticides, such as chlorpyrifos, poses a significant health risk in agricultural communities due to its harmful effects on the liver and nervous system. In northern Thailand, local populations traditionally use herbal remedies for detoxification, but scientific evidence supporting their efficacy is limited. This study evaluated a herbal detoxifying formula (Formula 04) containing *Thunbergia laurifolia* and *Embelia sessiliflora*, two medicinal plants traditionally used to alleviate pesticide-related symptoms. In vitro, the formula exhibited strong antioxidant activity and induced apoptosis in hepatic stellate cells, which are associated with liver fibrosis. In vivo, rats exposed to chlorpyrifos were treated with the formula using a traditional alternating-dose regimen over 18 days. Treated animals showed improved liver function, restoration of endogenous antioxidant defenses, reduced lipid peroxidation, and preservation of liver tissue architecture. No adverse effects or toxicity were observed. These results support the finding that this herbal formula is safe and effective for pesticide detoxification and provide scientific validation for its use in traditional Thai folk medicine.

## 1. Introduction

The liver is the primary organ responsible for maintaining metabolic homeostasis and detoxifying endogenous and exogenous compounds, including pesticides, drugs, alcohol, and environmental contaminants. Hepatic detoxification involves Phase I and Phase II biotransformation reactions. Phase I metabolism, catalyzed mainly by cytochrome P450 enzymes, converts lipophilic xenobiotics into reactive intermediates through oxidation, reduction, or hydrolysis; however, this process may simultaneously produce reactive oxygen species (ROS) that contribute to oxidative stress. Phase II reactions conjugate these metabolites with endogenous molecules such as glutathione (GSH), sulfate, or glucuronic acid to facilitate excretion through bile or urine [1]. When detoxification capacity is overwhelmed, excessive ROS generation leads to oxidative damage and hepatocellular injury.

Oxidative stress is a central mechanism of hepatotoxicity. Imbalance between ROS production and the endogenous antioxidant defense system causes mitochondrial dysfunction, lipid peroxidation, and DNA fragmentation [2]. The liver maintains redox homeostasis via enzymatic antioxidants such as superoxide dismutase (SOD), catalase (CAT), and glutathione peroxidase (GPx), as well as non-enzymatic antioxidants including reduced glutathione (GSH), vitamin C, and vitamin E [3,4]. However, chronic exposure to toxicants such as pesticides can exceed antioxidant capacity and impair hepatic function. Therefore, therapeutic strategies that enhance endogenous antioxidant defenses and promote detoxification are of significant interest.

Medicinal plants have been widely used for liver protection due to their antioxidant, anti-inflammatory, and detoxifying properties. Plant-derived hepatoprotective agents such as silymarin, curcumin, and gingerol have demonstrated efficacy in mitigating drug- and toxin-induced liver damage by modulating oxidative pathways and improving hepatic enzyme activity [5,6,7,8,9,10,11,12]. Polyherbal formulations are often superior to single-extract treatments due to synergistic phytochemical interactions that target multiple pathways associated with liver injury [13,14,15].

In northern Thailand, highland communities are regularly exposed to agricultural pesticides and traditionally use herbal remedies for detoxification. Our previous ethnopharmacological studies identified four indigenous plants with strong antioxidant and anti-pesticide properties: *Thunbergia laurifolia* Lindl., *Thunbergia coccinea* Wall. ex D. Don, *Morinda angustifolia* Roxb. var. *angustifolia*, and *Embelia sessiliflora* Kurz [16,17,18]. *T. laurifolia*. Their hepatoprotective and anti-inflammatory effects have been extensively documented [19,20,21,22,23,24], while *T. coccinea* has been shown to produce acetylcholinesterase (AChE) inhibitory activity [25]. *M. angustifolia* is traditionally used in jaundice and hepatitis management [26,27], and *E. sessiliflora* contains phenolic compounds with potent free radical scavenging activity [28,29].

These plants are rich in rosmarinic acid, caffeic acid, and other phenolic compounds that modulate oxidative and inflammatory signaling. In particular, these compounds activate the nuclear factor erythroid 2-related factor 2 (Nrf2) pathway, enhancing the expression of antioxidant enzymes such as heme oxygenase-1 (HO-1), CAT, and GPx, while downregulating NF-κB–mediated inflammation [30,31]. Despite their traditional use for detoxification, there is limited scientific validation of their combined hepatoprotective efficacy against pesticide-induced toxicity, especially under in vivo conditions.

Chlorpyrifos (CPF) is a widely used organophosphate pesticide that is still widely used in agricultural production, including in Southeast Asia, despite legal restrictions in several countries. In those areas, human exposure continues to occur through contaminated food and water as well as through occupational handling [32]. CPF induces hepatotoxicity through AChE inhibition, ROS overproduction, mitochondrial dysfunction, and inflammatory cell infiltration [33,34]. Due to ongoing environmental and occupational exposure risks, there is a critical need for safe and effective hepatoprotective interventions.

This study evaluated the antioxidant and hepatoprotective effects of a standardized detoxifying polyherbal formula (Formula 04) prepared from *T. laurifolia* and *E. sessiliflora*. Phytochemical profiling, in vitro antioxidant screening, and apoptosis assays using hepatic stellate (LX-2) cells were performed. In vivo hepatoprotective activity was investigated in chlorpyrifos-exposed rats by evaluating biochemical parameters (AChE activity, SOD, GSH, MDA), liver function enzymes, and histopathological changes. We hypothesized that Formula 04 would mitigate CPF-induced hepatotoxicity through redox regulation, enhancement of antioxidant defense, and attenuation of oxidative damage.

## 2. Materials and Methods

### 2.1. Preparation of the Plant Extract and Formula Quality Control

#### 2.1.1. Plant Collection

Four medicinal plants traditionally used for detoxification were selected based on ethnobotanical records from highland communities in Northern Thailand: *Thunbergia laurifolia* Lindl., *Thunbergia coccinea* Wall. ex D. Don, *Morinda angustifolia* Roxb. var. angustifolia, and *Embelia sessiliflora* Kurz. Fresh plant materials were collected from Ban Huai Sompoi, Mae Soi Subdistrict, Chom Thong District, Chiang Mai Province, Thailand. The botanical identity of all species was authenticated by a plant taxonomist at the Faculty of Pharmacy, Chiang Mai University. Voucher specimens were deposited in the Faculty of Pharmacy Herbarium under the following accession numbers: *T. laurifolia* (No. 0023428), *T. coccinea* (No. 0023429), *M. angustifolia* (No. 0023430), and *E. sessiliflora* (No. 0023431).

The parts used were leaves of *T. laurifolia* (TL), vines and leaves of *T. coccinea* (TC), roots of *M. angustifolia* (MA), and vines of *E. sessiliflora* (ES). After collection, the plant materials were washed, cut into small pieces, and oven-dried at 50 °C (Memmert, Schwabach, Germany) until moisture content was below 10%. The dried materials were then ground and passed through a 40-mesh sieve before extraction. The four single-herb extracts were included to establish their baseline biological activity. Seven additional polyherbal formulations were then prepared by systematically varying the relative proportions of the four herbs. These ratios were informed by documented traditional preparations used by highland communities where combinations of these herbs are routinely used for detoxification, as well as by principles of synergistic enhancement in Thai traditional medicine, which emphasize complementary actions achieved through specific herb pairings or mixtures. The percentage of each of the herbs used for developing the detoxification formulas is shown in Table 1.

#### 2.1.2. Preparation and Extraction

The powders of the individual herbs used in the detoxification formulations were extracted using the decoction method. Briefly, 500 g of powdered plant material was soaked in 7 L of distilled water for 30 min and then boiled for 2 h. The mixture was then filtered through cotton cloth and the residue was re-extracted twice under the same conditions using 5 L of water each time, followed by filtration through filter paper. All filtrates were combined and concentrated by gentle heating until reaching 3% Brix. Cab-o-sil (1.5% *w*/*v*) was then added, and the extract was spray-dried using a spray dryer (BUCHI, Bangkok, Thailand) to obtain a fine powder which was stored at 4 °C in airtight containers until use. The extraction yield was calculated as the percentage of dried extract compared to the initial amount of plant material. The resulting formulations and individual extracts were then evaluated for biological activity using antioxidant assays (DPPH and superoxide radical scavenging) and for their ability to induce apoptosis in human hepatic stellate cells (LX-2).

#### 2.1.3. Quality Control of Formula 04

The physical and chemical properties of the herbal formula were assessed using the official procedures of the THP 2018 [35].

##### Determination of Loss on Drying

Three grams of dried plant powder were placed into bottles which had been weighed beforehand. The samples were dried at 105 °C until their weight no longer changed, were cooled in a desiccator, and then re-weighed. The amount of weight lost was then calculated as a percentage.

##### Determination of Foreign Matter

After spreading 100 g of plant material thinly on a tray, foreign matter was visually separated and weighed. The percentage of foreign matter was then calculated.

##### Determination of Total Ash

After putting three grams of dried plant powder into pre-weighed crucibles, the samples were incinerated at 450 °C in an electric muffle furnace (Thermo Fisher, Waltham, MA, USA) until no carbon residue was obtained. The crucibles were then dried at 105 °C, cooled in a desiccator, weighed immediately, and the total ash content was calculated as a percentage.

##### Determination of Acid-Insoluble Ash

The total ash was treated with 25 milliliters of 2 M hydrochloric acid in the original crucibles, which were then covered with a watch glass and boiled for five minutes in a water bath. The insoluble matter was filtered using Whatman No. 40 paper (GE Healthcare, Chicago, IL, USA), rinsed with hot water until the filtrate became neutral, and then returned to the crucible. The samples were dried on a hot plate and subsequently incinerated at 500 °C an electric muffle furnace (Thermo Fisher, Waltham, MA, USA). The crucibles were then dried at 105 °C, cooled in a desiccator, then weighed immediately, and the percentage of acid-insoluble ash was determined. 

##### Determination of Ethanol-Soluble Extractive Value

Five grams of dried plant powder were macerated with 100 mL of 95% ethanol in a closed conical flask. The mixture was shaken for six hours, then allowed to stand for 18 h before the marc was rapidly filtered through Whatman No. 1 paper (GE Healthcare, Chicago, IL, USA). A 20 mL aliquot of the filtrate was evaporated to dryness in a pre-weighed dish using a water bath (Memmert, Schwabach, Germany). The extract was dried at 105 °C, cooled in a desiccator, and weighed immediately. Finally, the percentage of extractable matter was calculated.

##### Determination of Water-Soluble Extractive Value

After macerating five grams of dried plant powder with 100 mL of chloroform-water in a closed conical flask, the mixture was shaken for six hours and then allowed to stand for 18 h. The marc was rapidly filtered using Whatman No. 1 paper. A 20 mL aliquot of the filtrate was evaporated to dryness in a pre-weighed dish using a water bath. The extract was dried at 105 °C, cooled in a desiccator, and weighed immediately. The content of extractable matter was then calculated and expressed as a percentage.

### 2.2. Compact Mass Spectrometry (CMS) of Formula 04

The solution of Formula 04 (0.01 mg/mL in 99% methanol, HPLC grade) was filtered through a 0.22 µm syringe filter. The mobile phase consisted of 0.01% (*v*/*v*) formic acid in acetonitrile, delivered at a flow rate of 0.2 mL/min. A 10 µL aliquot was directly injected into a compact mass spectrometer (CMS; Advion Interchim Scientific, Ithaca, NY, USA). Mass spectral data were acquired in selected ion monitoring (SIM) mode using a positive atmospheric pressure chemical ionization (APCI) source (resolution within 0.5–0.7 *m*/*z*) and were compared with standards of rosmarinic acid (*m*/*z* 361.318 [M + H]^+^).

### 2.3. Antioxidant Activity Study

The antioxidant activities of the individual extracts and the detoxifying formula were evaluated using two assays: DPPH radical scavenging and superoxide radical scavenging.

#### 2.3.1. DPPH Radical Scavenging Activity Assay

DPPH radical scavenging is a test for the antioxidant activity of various substances using the 2,2-diphenyl-1-picrylhydrazyl radical, a highly stable synthetic radical which gives a purple color in methanol solution. DPPH radicals can react with antioxidant substances, causing the transfer of hydrogen atoms from antioxidants to DPPH radicals, resulting in a decrease in the purple color of the DPPH solution or a change to a yellow color. The experiment was performed by preparing the test substances in various concentrations, adding 20 µL of sample to a 96-well plate and 180 µL of DPPH reagent (90 µM), mixing well and keeping it in the dark at room temperature for 30 min. Absorbance was measured at 517 nm by a microplate reader (BioTek Instruments, Winooski, VT, USA). Deionized water was used as the blank and gallic acid was the standard in this assay [36,37]. The percentage of DPPH scavenging activity was calculated using the following formula as Equation (1):

(1) $$DPPH\;scavenging\left(\%\right)=\frac{Absorbance\;of\;control-Absorbance\;of\;test\;sample}{Absorbance\;of\;control}\times 100$$

IC_50_ values, defined as the concentration of sample required to inhibit 50% of DPPH radicals, were obtained using linear regression analysis and served as a measure of the extract’s antioxidant activity.

#### 2.3.2. Superoxide Radical Scavenging Activity Assay

This assay is used to evaluate the ability of a compound (often a potential antioxidant) to scavenge superoxide radicals (O_2_^•^)^−^ which are reactive oxygen species (ROS) produced in various biological systems. The superoxide radical is highly reactive and can cause damage to cells, proteins, lipids, and DNA. For that reason, the ability to scavenge superoxide radicals is an important indicator of a compound’s potential antioxidant properties [38,39]. All reagents used in the study were prepared in 0.1 M phosphate buffer (pH 7.4). An equal volume of 50 µL of NBT (0.2 mM), NADH (0.5 mM), and various concentrations of sample were pipetted into 96-well plates and mixed. Then 50 µL of PMS (25 µM) solution was added and mixed. The reaction mixture was incubated for 15 min at room temperature then the absorbance was measured at 560 nm. The experiment was performed three times. Gallic acid was used as a standard. Superoxide radical scavenging percentages and IC_50_ values were determined using the same equation applied for the DPPH assay.

### 2.4. Study of the Effect of Test Substances on Cultured Liver Cells

The cytotoxic assay and apoptosis-inducing potential of Formula 04 was evaluated using human hepatic stellate cells (LX-2), which are commonly used to model hepatic fibrosis and toxic injury.

#### 2.4.1. Cell Line and Cell Culture

LX-2 cells (Merck Millipore, Billerica, MA, USA) were cultured in Dulbecco’s Modified Eagle Medium (DMEM; Gibco ^TM^, Waltham, MA USA) supplemented with 2% fetal bovine serum (FBS), 2 mM l-glutamine, 100 U/mL penicillin, and 100 µg/mL streptomycin. The cells were incubated at 37 °C in CO_2_ incubator. Culture medium was replaced every three days. Once the cells reached 70–80% confluence, they were harvested, plated, or sub-cultured for storage or for use in subsequent passages.

#### 2.4.2. Cell Viability Assay

LX-2 cells were seeded into 96-well plates (1 × 10^4^ cells/well) and incubated at 37 °C with 5% CO_2_ in the incubator for 24 h. Then various concentrations of the extracts were added to the cells and further incubated for either 24 or 48 h. Cell viability was detected using a Sulforhodamine B (SRB) colorimetric assay [40,41]. Briefly, cold TCA (10%, *w*/*v*) was added to the wells and the plate was kept at 4 °C for 1 h to fix the cells. Then the plate was washed with tap water and dried after which the cells were stained with 0.057% SRB at room temperature for 30 min. The excess dye was then washed off using acetic acid (1%, *v*/*v*) and the cells were allowed to dry. Finally, Tris’s base solution (10 mM) was added to dissolve the dye, and the absorbance was measured using a microplate reader (BioTek Instruments, Winooski, VT, USA) at a wavelength of 510 nm.

#### 2.4.3. Apoptosis Assay

Briefly, LX-2 cells were seeded in 6-well plates at a density of 3 × 10^5^ cells/well and incubated at 37 °C in a 5% CO_2_ incubator for 24 h. After that period, the cells were treated with various concentrations of the test substances and incubated for an additional 24 h. Subsequently, the cells were washed with cold PBS and detached using trypsin. The cell suspension was centrifuged at 1200 rpm for 5 min, washed twice with cold PBS, and the supernatant was discarded. The pellet was resuspended in 90 µL of binding buffer and kept on ice. Five µL each of Annexin V and propidium iodide (ImmunoTools, Friesoythe, Germany) were then added and mixed gently. The samples were incubated in the dark for 20 min, followed by the addition of 400 µL of binding buffer. Apoptotic and necrotic cells were subsequently analyzed by flow cytometry (Beckman Coulter CyAn ADP; Beckman Coulter, Brea, CA, USA) [42].

### 2.5. Anti-Pesticide Potential of Detoxifying Formulas in Experimental Animals

#### 2.5.1. Experimental Animals

Four-week-old male Sprague–Dawley rats (180–200 g) were obtained from Nomura Siam International Co., Ltd. (Bangkok, Thailand) and maintained in a temperature-controlled room (25 ± 1 °C) under a 12 h light/dark cycle. Animals had ad libitum access to standard laboratory chow and water and were acclimatized for seven days before the experiment. Ethical approval for animal use (Approval No. 49/2559) was obtained from the Ethics Committee on the Use of Experimental Animals, Faculty of Medicine, Chiang Mai University, Thailand.

For the in vivo study, thirty rats were randomly divided into five groups (*n* = 6 per group) [43]. Formula 04 was administered to all rats orally once daily for 18 consecutive days, 30 min before CPF administration [44,45,46,47].

The treatment groups were assigned as follows:

Group 1 Normal control: received distilled water only
Group 2 CPF control: received CPF (16 mg/kg BW)
Group 3 F04 low dose: received Formula 04 in a cyclic dosing regimen of 200, 100, and 30 mg/kg BW on alternating days
Group 4 F04 medium dose: received Formula 04 in a cyclic dosing regimen of 400, 200, and 60 mg/kg BW on alternating days
Group 5 F04 high dose: received Formula 04 in a cyclic dosing regimen of 800, 400, and 120 mg/kg BW on alternating days

This cyclic dose schedule (6 cycles over 18 days) was designed to mimic traditional Thai detoxification practices. At the end of the experimental period, rats were fasted for 16–18 h and anesthetized with pentobarbital sodium (50 mg/kg BW, intraperitoneally). Blood and organs were collected for biochemical and histopathological analysis.

#### 2.5.2. Acetylcholinesterase (AChE) Activity Assay

The principle of AChE activity assay is based on the enzyme’s ability to hydrolyze the substrate acetylcholine (ACh) into two products: acetate and choline [48]. In the presence of a specific reagent like DTNB (5,5′-dithiobis-2-nitrobenzoic acid), the reaction generates a measurable color change, which is directly related to the level of enzyme activity. In this study, an AChE activity assay was conducted using a Sigma-Aldrich assay kit (Sigma-Aldrich, St. Louis, MO, USA), following the manufacturer’s instructions. To conduct the experiment, 10 µL of diluted whole blood samples were dispensed into a 96-well plate. Subsequently, 190 µL of the working reagent was added to each well and mixed thoroughly. The reaction mixtures were incubated at room temperature, and absorbance was measured at 2 min and 10 min using a microplate reader (BioTek Instruments, Winooski, VT, USA) set to 412 nm. AChE activity was calculated according to Equation (2):

(2) $$AChE\;activity\;\left(units/L\right)=\frac{A\left(sample\right)\;at\;10\;min-A\left(sample\right)\;at\;2\;min}{A\left(calibrator\right)\;at\;10\;min-A\left(blank\right)\;at\;10\;min}\times n\times 200$$

where

A = absorbance
200 = equivalent activity (units/L) of the calibrator when assayed is read at 2 and 10 min
n = dilution factor

#### 2.5.3. Oxidative Stress Biomarkers Determination

Measuring oxidative stress biomarkers in serum provides valuable insights into the balance between oxidants and antioxidants in the rat’s body. In this study, kits for oxidative stress biomarkers, including malondialdehyde (MDA; Elabscience^®^, Houston, TX, USA), reduced glutathione (GSH; Sigma-Aldrich), and superoxide dismutase (SOD; Randox Lab, Crumlin, UK), were used to investigate oxidative stress in the rats.

#### 2.5.4. Hematology and Blood Chemistry Analysis

Blood components including red blood cells (RBC), white blood cells (WBC), hemoglobin (HGB), hematocrit (HCT), platelets (PLT), mean corpuscular hemoglobin (MCH), mean corpuscular volume (MCV), mean corpuscular hemoglobin concentration (MCHC), neutrophils (Nu), lymphocytes (Lymph), monocytes (Mono), eosinophils (E), and basophils (Ba) were investigated using a Mindray BC-5300 Vet automated hematology analyzer (Shenzhen, China). 

Blood biochemical parameters, including blood urea nitrogen (BUN), creatinine (Cr), total protein (TP), albumin (ALB), total bilirubin (TB), direct bilirubin (DB), aspartate aminotransferase (AST), alanine aminotransferase (ALT), and alkaline phosphatase (ALP), were determined using an automated BX-3010 analyzer (Sysmex, Kobe, Japan).

#### 2.5.5. Histological and Pathological Examination

The rat livers and kidneys were harvested, weighed, and then fixed in 10% formalin. After fixation, the tissues were dehydrated using a graded ethanol series, followed by replacement of ethanol with a solvent (such as xylene) to make the tissue transparent. The tissues were then embedded in paraffin. The paraffin blocks were sectioned to a thickness of 4 μm, stained with hematoxylin and eosin (H&E), and observed under a microscope (Olympus CX-23; Olympus Corporation, Tokyo, Japan).

### 2.6. Statistical Analysis

Results are expressed as mean ± S.E.M. Statistical analyses were performed using GraphPad Prism version 8 (GraphPad Software, San Diego, CA, USA). Initially, the Shapiro–Wilk test was used to assess the normality of the data. If the data did not significantly deviate from a normal distribution, ANOVA was followed by Tukey’s multiple comparison tests. If the Shapiro–Wilk test indicated a non-normal distribution, the Kruskal–Wallis nonparametric ANOVA test followed by Dunn’s test were applied. Statistical significance was set at *p* < 0.05.

## 3. Results

### 3.1. Preparation of Plant Extracts and Quality Control of Formula 04

The quality of the raw materials, *T. laurifolia* (TL), *T. coccinea* (TC), *M. angustifolia* (MA), and *E. sessiliflora* (ES), was evaluated by comparing the obtained data with the previously reported specifications for each plant. Morphological characteristics were noted. Organoleptic tests and microscopic examinations were also conducted. The tissues of each plant were observed and analyzed for consistency with previously documented information [44]. The four herbs were formulated by adjusting the types and quantities based on local wisdom. The different detoxifying formulas, designated as Formulas 01–07, were extracted by decoction with water and then spray dried to obtain a brownish-yellow powder. The extraction yields of Formulas 01–07 were 16.25, 16.19, 15.75, 17.20, 19.15, 19.34, and 17.75, respectively.

**Figure 1:**
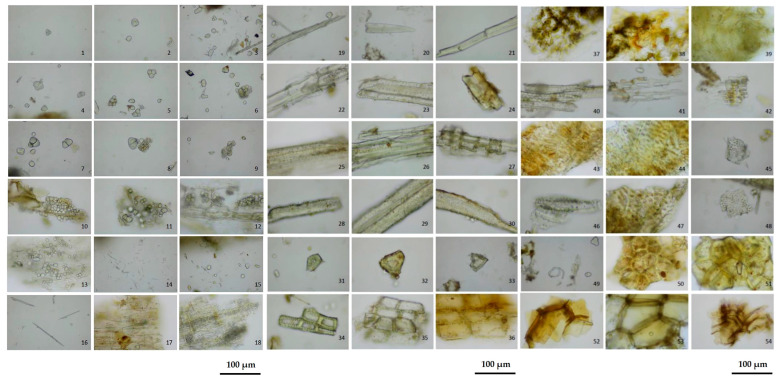
Representative microscopic features observed in the powdered detoxifying Formula 04. Key plant tissue structures include: (1–9) starch grains; (10–13) parenchyma with starch grains; (14–16) acicular crystals; (17–18) parenchyma with acicular crystals; (19–26) fiber types; (27) medullary ray; (28–30) fibers with prismatic crystals; (31–33) stone cells; (34–36) macrosclereids; (37) chlorenchyma; (38) wavy epidermis; (39) epidermis with stomata; (40) tracheids; (41–43) reticulated vessels; (44–48) bordered-pitted vessels; (49) spiral vessel; (50–51) phelloderm; and (52–54) cork cells.

Activity tests were conducted on Formulas 01–07. The results of this comprehensive analysis indicated that Formula 04 exhibited the highest level of activity. Following that, Formula 04 was assessed for its physicochemical properties according to the official methods outlined in the Thai Herbal Pharmacopoeia 2018 [35]. The microscopic characteristics of the powdered drug are illustrated in Figure 1.

The physicochemical examinations of the crude drug included loss on drying, total ash, acid-insoluble ash, ethanol-soluble extractive value, and water-soluble extractive value. (Mean values are presented in Table 2). Formula 04 was prepared using a traditional water-based decoction method. However, the specifications for this detoxification formula are not documented in any pharmacopeia or textbook. This study defined the specifications for Formula 04 in accordance with the methods outlined in the Thai Herbal Pharmacopoeia. By implementing these practices, our aim was to standardize quality control for future studies that involve these raw materials.

Preliminary testing of the crude extract using this formula revealed positive results for phenolic and flavonoid groups. Thin-layer chromatography (TLC) was used to determine the chemical profile of Formula 04 compared with the individual herbs, TL and ES.

### 3.2. Chemical Composition by Compact Mass Spectrometry (CMS) of Formula 04

**Figure 2:**
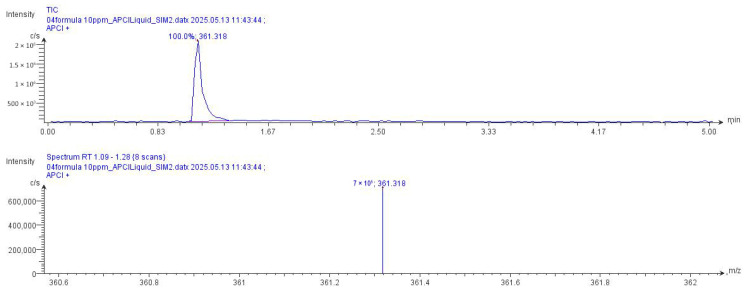
Atmospheric-pressure chemical ionization (APCI) base peak chromatogram and mass spectrum of a Rosmarinic acid in the 04 recipe extract, with positive ion selected ion monitoring (SIM) mode at 361.318 *m*/*z* ([M + H]^+^).

**Figure 3:**
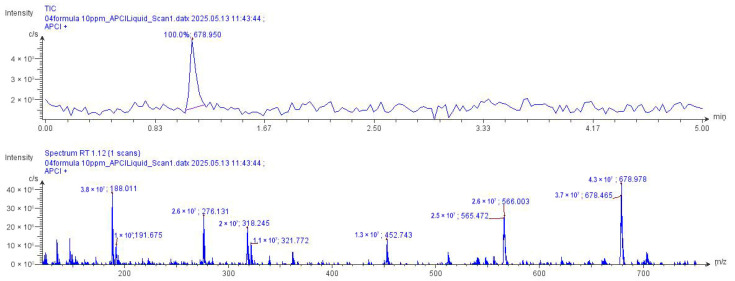
Atmospheric-pressure chemical ionization (APCI) mass spectrum of the 04-recipe extract with positive ion scan mode at 10–1200 *m*/*z*.

CMS analysis successfully identified a wide range of compounds in individual herbal extracts and complex herbal formulations. Rosmarinic acid, used as a marker compound, was detected in all tested samples, confirming its presence in both single-plant extracts and multi-herbal recipes (Figure 2 and Figure 3). Notably, the herbal formulations, particularly Recipes 04, ES 01_V, and TL 01_L, exhibited strong signals for rosmarinic acid along with several other high-intensity compounds. These results suggest that the combined herbal extracts possess complex synergistic phytochemical profiles that may enhance biological activity. The consistent detection of rosmarinic acid across all samples validates the analytical method and supports its use as a standard marker for quality control in herbal analysis.

### 3.3. Screening of Antioxidant Activity

**Figure 4:**
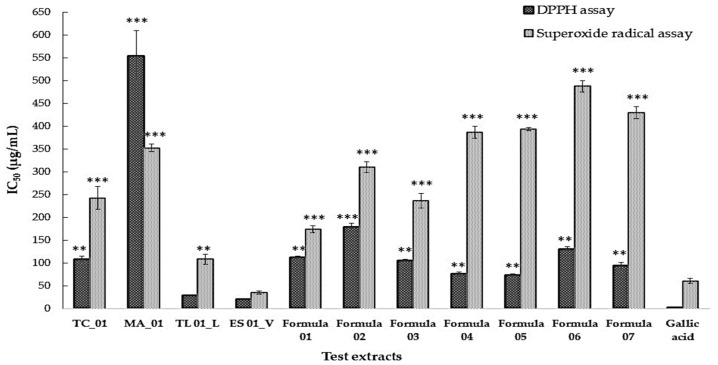
Comparison of IC_50_ values of detoxifying formulas based on DPPH radical scavenging and superoxide radical scavenging assays. Results are presented as mean ± S.E.M. (*n* = 3). Significant differences compared to gallic acid are indicated as: ** *p* < 0.01, *** *p* < 0.001.

*E. sessiliflora* (ES) and *T. laurifolia* (TL) exhibited the strongest antioxidant activity in both the DPPH and superoxide radical assays, followed by *T. coccinea* (TC) and *M. angustifolia* (MA). Among the detoxification formulas, Formulas 04 and 05 showed the highest antioxidant activity in the DPPH assay, with IC_50_ values of 76.0 ± 4.9 and 74.3 ± 2.7 µg/mL, respectively, compared to gallic acid, the reference standard, which yielded an IC_50_ of 2.7 ± 0.52 µg/mL. In the superoxide radical scavenging assay, Formulas 01 and 03 demonstrated greater scavenging potential than the other formulas (Figure 4).

Based on these results, test extracts TL 01_L, ES 01_V, and Formulas 01, 03, 04, and 05 were selected for further investigation.

### 3.4. Cytotoxic and Apoptotic Effects of Test Substances in Cultured Liver Cells

#### 3.4.1. Effects of Test Substances on Cell Viability

**Figure 5:**
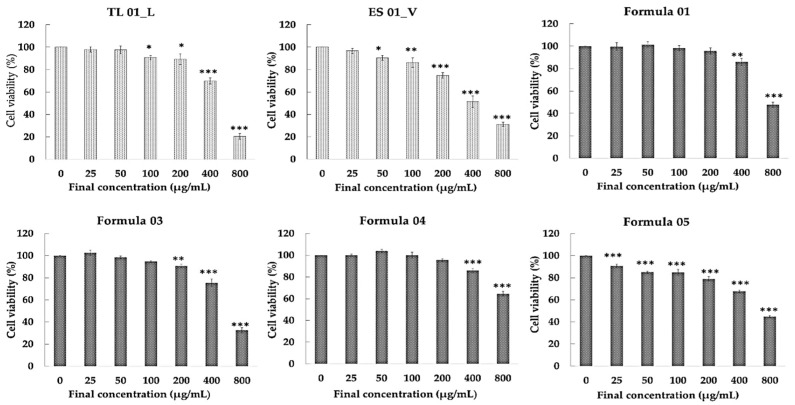
Effect of detoxifying formulas on viability of LX-2 cells after 24 h treatment. Data are expressed as mean ± S.E.M. (*n* = 3). * *p* < 0.05, ** *p* < 0.01, *** *p* < 0.001 compared to untreated control cells.

The impact of test extracts and detoxification formulas on the viability of LX-2 cells is presented in Figure 5. The results indicate that Formula 01, and Formula 04 induced statistically significant cell death at concentrations of 400–800 μg/mL. Formula 03 caused significant cell death at 200–800 μg/mL. The ES_01_V extracts demonstrated cell death at concentrations ranging from 50 to 800 μg/mL. Additionally, the TL 01_L and Formula 05 caused significant cell death at concentrations of 100–800 μg/mL and 25–800 μg/mL, respectively.

Based on this assay, the three concentrations that caused significant cell death were selected for the apoptosis assay.

#### 3.4.2. Apoptotic Effects of Formula 04

**Figure 6:**
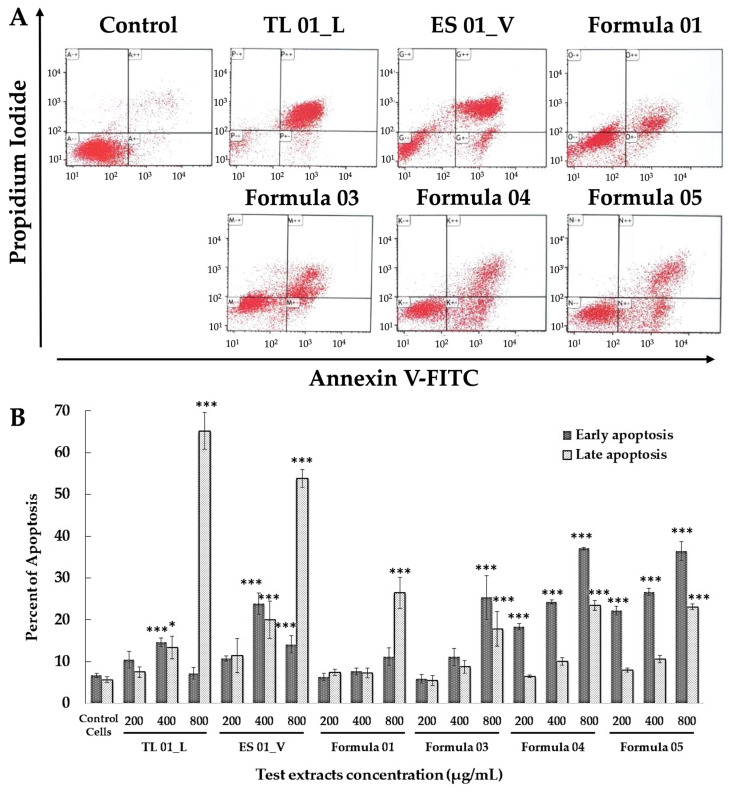
Flow cytometric analysis of apoptosis induction in LX-2 cells treated with detoxifying formulas at 200–800 μg/mL. (**A**) Dot plots representing Annexin V/PI staining; (**B**) Bar graph summarizing early and late apoptosis. Data shown as mean ± S.E.M. (*n* = 3). * *p* < 0.05, *** *p* < 0.001 vs. control cells.

The effects of the detoxifying formulas on apoptotic cell death are shown in Figure 6. The results indicate that the highest concentrations of TL 01_L and ES 01_V significantly induced late apoptosis compared to the control cells, suggesting these extracts were highly toxic, with minimal induction of early apoptosis. Interestingly, Formulas 04 and 05 were particularly effective in promoting early apoptosis, with the percentage of early apoptosis increasing at higher concentrations.

Among the detoxifying formulas, Formulas 04 and 05 exhibited a higher percentage of early apoptosis and a lower percentage of late apoptosis compared to the individual herbal extracts. Formula 04 demonstrated superior antioxidant activity and apoptosis induction compared to Formula 05. As a result, Formula 04 was selected for further in vivo studies.

### 3.5. Effects of Formula 04 on Pesticide-Induced Toxicity in Experimental Animals

#### 3.5.1. Rat Body Weight and Rat Organ Weight Changes

The rats used in the anti-pesticide test were examined and evaluated in various aspects such as respiratory rate and breathing pattern, gastrointestinal and excretory systems, nervous system, skeletal muscle system, and observed behavior, including surveillance for diseases in the experimental animals and examination of overall health. The results indicate that all rats remained healthy, showing no signs of abnormalities, illness, or unusual symptoms throughout the study. The initial and final body weights of the rats are summarized in Table 3. The CPF control group exhibited significant weight loss by Day 18 compared to the normal control group, reflecting substantial toxicity. In contrast, rats treated with Formula 04 demonstrated recovery of body weight toward baseline levels.

After 18 days of treatment with the detoxifying Formula 04 extract, liver and kidney tissues were collected and weighed, as shown in Table 4. The results revealed no significant differences in liver and kidney weights between rats treated with any of the three forms of Formula 04 and the control group receiving distilled water with pesticide.

#### 3.5.2. Effect of Formula 04 Extract on Acetylcholinesterase Enzyme and Antioxidant Biomarkers

**Figure 7:**
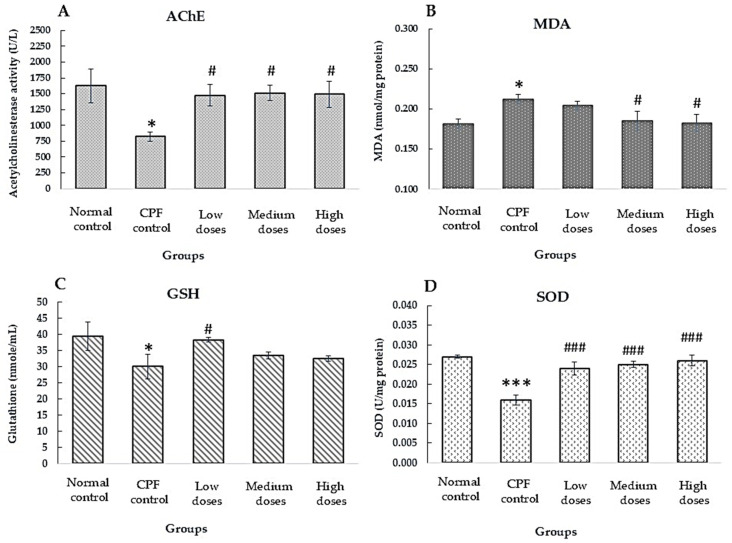
Effect of Formula 04 on acetylcholinesterase (AChE) activity and antioxidant biomarkers in chlorpyrifos-exposed rats. (**A**) AChE enzyme activity; (**B**) malondialdehyde (MDA) levels; (**C**) glutathione (GSH) levels; and (**D**) superoxide dismutase (SOD) activity. Data are presented as mean ± S.E.M. (*n* = 6). * *p* < 0.05, *** *p* < 0.001 vs. normal rats; ^#^ *p* < 0.05, ^###^ *p* < 0.001 vs. control (pesticide-only) group.

The overall findings suggest that Formula 04 extract has potential to counteract pesticide toxicity, primarily by enhancing acetylcholinesterase activity. Additionally, it inhibits free radical formation by boosting antioxidant enzyme levels (including SOD), increasing glutathione levels, and reducing lipid peroxidation by lowering MDA. Notably, at doses of 800, 400, and 120 mg/kg, Formula 04 produced enzyme activity values (GSH, AChE, and SOD) comparable to those observed in normal rats receiving only distilled water (Figure 7). These high doses (800, 400, and 120 mg/kg) appear suitable for further long-term toxicity studies.

#### 3.5.3. Impact of Formula 04 Extract on Hematological Parameters

The findings indicate that control rats treated with distilled water and pesticides showed a significant increase in RBC, HGB, MCV, MCH, WBC, Nu, Lymph, and Ba compared to normal rats (*p* < 0.05), as shown in Table 5. Rats administered low doses of Formula 04 (200, 100, and 30 mg/kg body weight) exhibited significantly reduced levels of WBC, Nu, Lymph, and Ba compared to the control group (*p* < 0.05). Similarly, rats receiving middle doses of Formula 04 (400, 200, and 60 mg/kg body weight) demonstrated a significant decrease in WBC, Nu, and Ba compared to the control group (*p* < 0.05). In the group treated with high doses of Formula 04, a reduction in Nu and Ba was observed. Notably, some hematological parameters in rats treated with Formula 04 were comparable to those in normal rats. The observed changes in values were not significant enough to indicate abnormalities because all abnormal values were still within the normal range.

#### 3.5.4. Impact of Formula 04 Extract on Blood Chemistry Profile

The blood chemistry results are presented in Table 6. Rats in the control group, which received distilled water and pesticides, displayed significantly elevated levels of AST enzyme compared to the normal rats (*p* < 0.05). In contrast, treatment with Formula 04 extract at low, middle, and high doses resulted in significantly lower AST enzyme levels than those observed in the control group (*p* < 0.05). Furthermore, rats treated with middle and high doses of Formula 04 extract exhibited slight but significant reductions in BUN levels. The data indicate that rats treated with Detoxifying Formula 04 showed blood chemistry values comparable to those of the normal rats. The observed changes in these values were minimal and insufficient to suggest any significant abnormalities that would have required confirmation through pathological analysis.

#### 3.5.5. Effects of Formula 04 on Chlorpyrifos-Induced Liver Histopathology

**Figure 8:**
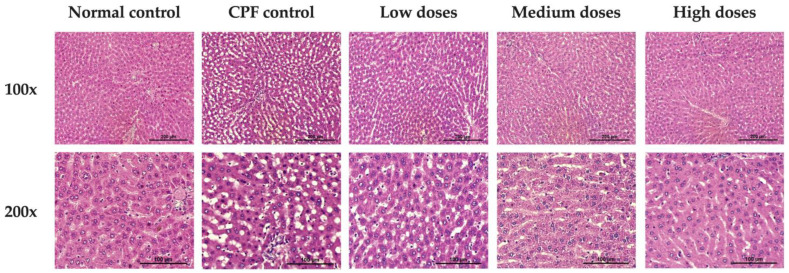
Histopathologic results of evaluation of rat livers in the normal group, the control group, and the Formula 04-treated group which received three doses of Formula 04.

**Figure 9:**
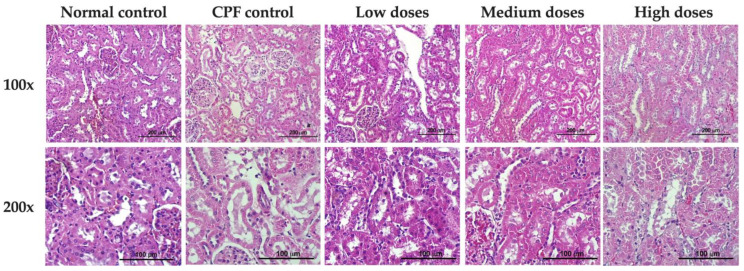
Histopathologic results of rat kidneys in the normal group, control group, and the Formula 04-treated group which received three doses of Formula 04.

Normal control exhibited healthy liver and kidney tissues. In contrast, CPF control that received distilled water and pesticide showed significant liver abnormalities, including mild to moderate steatosis and fatty liver and dilation in sinusoids, which were notably different from those of the normal control. However, their kidneys appeared histologically normal. In rats treated with Detoxifying Formula 04 extract, fat accumulation in the liver was observed; however, increasing the extract dose significantly reduced the extent and severity of these changes. Rats in the test groups that received high doses of the extract (800, 400, and 120 mg/kg) showed liver and kidney tissues most like those of the normal control (Figure 8 and Figure 9).

## 4. Discussion

Pesticide-induced liver fibrosis is a progressive condition resulting from chronic xenobiotic exposure which can lead to hepatocyte injury, persistent inflammation, and excessive deposition of extracellular matrix proteins, particularly collagen. These changes disrupt hepatic architecture and may progress to cirrhosis. Mechanistically, pesticides promote the release of pro-inflammatory cytokines such as TNF-α and IL-6, activate HSCs, and generate reactive metabolites that exacerbate oxidative stress and tissue damage [49,50]. Epigenetic regulation also contributes to the activation of fibrogenic pathways. Therapeutic strategies thus aim to reduce oxidative stress, inhibit HSC activation, and attenuate TGF-β/Smad signaling [51]. Pesticide-induced AChE inhibition drives neurotoxicity and systemic injury; however, antioxidant-rich herbs can mitigate this damage and improve long-term health. Natural products with antioxidant, anti-inflammatory, and pro-apoptotic properties are of particular interest, with nutraceutical antioxidants—including vitamins, flavonoids, and carotenoids—capable of counteracting ROS and modulating apoptosis-related pathways [52,53,54,55,56,57]. Screening plant extracts for these activities not only validates traditional use but also provides molecular leads for novel antifibrotic interventions.

In this study, seven combinations of four plant extracts were evaluated for antioxidant activity and apoptosis induction in LX-2 cells. In the DPPH assay, antioxidant activity ranked Formula 04 > 05 > 07 > 03 > 01 > 06 > 02, whereas the superoxide radical scavenging assay ranked Formula 01 > 03 > 02 > 04 > 05 > 07 > 06. Based on these results, Formulas 01, 03, 04, and 05 were selected for apoptosis analysis. Among them, Formula 04—comprising *T. laurifolia* (TL) and *E. sessiliflora* (ES) in a 1:1 ratio—demonstrated the strongest overall effect, particularly in early apoptosis induction. Phytochemical screening demonstrated that Formula 04 is enriched in phenolic and flavonoid compounds. *T. laurifolia* is widely recognized for its antioxidant and detoxifying properties, including reduction in ROS, enhancement of endogenous antioxidant enzyme systems, and protection against xenobiotic- or glutamate-induced apoptosis, as demonstrated in both preclinical and clinical studies [20,58,59]. In contrast, *E. sessiliflora* has been less extensively studied, but members of its genus possess strong antioxidant capacity derived primarily from phenolic compounds and flavonoids, with documented antioxidant and pro-apoptotic effects [60,61,62]. The effects of rosmarinic acid and caffeic acid are consistent with their well-documented antioxidant and hepatoprotective activities. Previous studies have shown that rosmarinic acid exhibits stronger antioxidant activity than caffeic acid [63,64]. In the present study, CMS analysis confirmed the presence of rosmarinic acid in Formula 04, supporting its potential to exert antioxidant effects.

Apoptosis, or programmed cell death, is a tightly regulated mechanism that removes damaged, infected, or cancerous cells without provoking inflammation, thereby maintaining tissue homeostasis. In the liver, apoptosis removes dysfunctional or aged cells and prevents the accumulation of harmful or pre-cancerous cell populations. Under normal conditions, antioxidants protect hepatocytes from oxidative stress–induced apoptosis, whereas in pathological states such as fibrosis or cancer, they can modulate signaling pathways (e.g., p53, NF-κB) to promote apoptosis and limit disease progression [55]. Antifibrotic strategies often target these pathways by preventing HSC activation, inducing apoptosis in activated HSCs, or promoting their deactivation, which can reverse fibrosis and support liver recovery [65].

In this study, apoptosis was evaluated in LX-2 cells, a well-established antifibrotic model [66]. Formula 04 preferentially induced early apoptosis with minimal progression to late apoptosis, whereas TL and ES extracts alone predominantly triggered late apoptosis, a pattern associated with higher cytotoxicity [67]. Mechanistically, TL-derived compounds such as rosmarinic modulate caspase-3/7 activity and preserve mitochondrial integrity, thereby protecting hepatocytes from ROS-mediated cell death [68,69]. ES contains XIAP-inhibiting compounds that promote caspase-dependent apoptosis in hepatoma and HSC models [70,71]. Together, these complementary actions favor early apoptosis, selectively eliminating fibrogenic HSCs while preserving hepatocytes. Consistent with previous reports that polyphenols and flavonoids mitigate oxidative stress while selectively triggering apoptosis in damaged or malignant hepatocytes [72,73], these results suggest synergistic effects of TL and ES specifically, TL provides antioxidant protection, while ES facilitates apoptosis in activated HSCs and supports antioxidant enzyme recovery. This dual action underlies the observed in vitro antifibrotic potential of Formula 04. In addition, apoptosis testing also reflected in vitro safety.

The significance of Formula 04 clarifies and expands on existing literature. In our study, Formula 04 exhibited a distinct early-apoptosis profile in LX-2 cells, consistent with reports that phenolic-rich extracts promote controlled apoptotic clearance of activated HSCs while minimizing nonspecific cytotoxicity [73]. This pattern supports a synergistic interaction between TL and ES, aligning with evidence that combining antioxidant-dominant herbs with pro-apoptotic constituents enhances antifibrotic efficacy [73]. Such synergy is mechanistically plausible as TL-derived phenolics help maintain cellular redox balance [58,59], whereas ES-derived compounds promote caspase-dependent apoptosis [70,71]. Similar complementary actions have been reported in other polyherbal antifibrotic formulations [60,61,62]. The Formula 04 was largely non-cytotoxic across the tested range. Only the highest concentration (800 µg/mL) produced a modest rise in late apoptosis, suggesting a threshold at which nonspecific cytotoxicity may begin. This indicates that the pro-apoptotic activity at lower, effective concentrations (100–400 µg/mL) is likely a selective, mechanism-driven effect rather than general toxicity. However, to demonstrate the safety of this formulation for potential human use, pre-clinical evaluation must include standardized single-dose and repeated-dose toxicity studies in experimental animals, in accordance with standard guidelines [74,75,76,77].

Body weight and organ weight are primary parameters that indicate abnormalities in animals exposed to continuous organophosphate administration. In this study, rats receiving chlorpyrifos at a dose of 16 mg/kg BW exhibited a significant reduction in body weight compared with the normal control group. Weight reduction is a well-documented outcome in sub-chronic or sub-acute CPF exposure, associated with cholinergic stress, gastrointestinal disturbance, and metabolic imbalance in rodent models. The normalization of body weight in CPF-exposed rats treated with the herbal formula suggests that the formula exerts protective, antioxidant, and hepatoprotective effects that mitigate cholinergic and oxidative stress–related metabolic disturbances induced by CPF [78,79]. However, the liver and kidney weights were not different from the normal rats which may indicate that the 18-day exposure period was not long enough to induce detectable organ abnormalities.

Chlorpyrifos exposure induced hallmark features of hepatotoxicity, including suppressed AChE activity, elevated MDA, reduced SOD and GSH, hematological disturbances, and increased AST and ALT. Formula 04 (800, 400, and 120 mg/kg) reversed these effects by restoring AChE activity, enhancing antioxidant defenses, normalizing red blood cell indices, and lowering AST and BUN with greater efficacy at medium and high doses. The restoration of AChE activity in CPF-exposed rats treated with Formula 04 is particularly noteworthy. Because organophosphate-induced AChE inhibition is largely irreversible due to phosphorylation and aging of the enzyme, the observed recovery is more likely attributable to de novo synthesis of newly formed AChE, rather than direct reactivation of the inhibited enzyme [45,46]. The antioxidant constituents of Formula 04—particularly rosmarinic acid—may mitigate lipid peroxidation and protein carbonylation, thereby protecting the structural integrity and functionality of regenerated AChE [79,80].

GSH, SOD, and MDA are key biomarkers associated with oxidative processes. During oxidative stress, MDA levels typically increase, whereas GSH and SOD levels decrease. In this study, Formula 04 elevated GSH and SOD levels while reducing MDA levels. Previous reports have demonstrated that rosmarinic acid can exert similar antioxidant effects; therefore, the activity observed in Formula 04 is likely attributable, at least in part, to the presence of rosmarinic acid [80,81,82].

This experiment employed a high dose of organophosphate administered over a short duration, with the objective of only inducing hepatic injury. Although some hematological parameters showed statistically significant differences, all values remained within the normal physiological range. Thus, it may be inferred that exposure to chlorpyrifos at 16 mg/kg for 18 days did not cause overt dysfunction of hematopoietic organs. Importantly, chlorpyrifos-exposed rats exhibited elevated AST levels. Administration of Formula 04 restored AST values to within normal limits. This hepatoprotective effect may be explained by the antioxidant activity of rosmarinic acid present in the formulation, which likely mitigated chlorpyrifos-induced hepatocellular damage, thereby reducing AST levels [32,82,83].

CPF has been associated with both hepatic and renal histopathological alterations in rodent studies [45,46]. In the present study, histological examination confirmed preservation of hepatic architecture and narrowing of hepatic sinusoids in treated animals, with high-dose groups appearing most similar to the normal controls. In contrast, no renal lesions were observed in chlorpyrifos-exposed rats. This outcome is consistent with previous reports indicating that CPF-induced renal injury is both dose- and exposure-dependent [84], and the duration of exposure in the current study may not have been sufficient to induce detectable renal pathology.

Importantly, Formula 04 did not induce renal pathology at any dose, underscoring its renal safety. These findings are consistent with previous reports showing that rosmarinic acid [80,85,86], as well as extracts of *T. laurifolia* [79] and *Embelia* species [87] and *Embelia* species [88], protect against toxin-induced renal injury through antioxidant and anti-inflammatory mechanisms. Rosmarinic likely contribute to these effects by elevating hepatic GSH, suppressing lipid peroxidation, and attenuating inflammatory signaling, thereby reducing hepatocellular injury and fibrosis [81,82,83,89]. Their combined presence in Formula 04 may therefore offer additive protection against oxidative and inflammatory stressors. It is well-established that rosmarinic acid can increase the expression of the Nrf2 gene, and that the upregulation of this gene can attenuate the development of liver fibrosis [32,89]. Collectively, these protective actions likely reflect a convergence of ROS scavenging, Nrf2-mediated antioxidant activation, and apoptosis of activated hepatic stellate cells [80,90].

## 5. Conclusions

Among the seven formulations evaluated, Formula 04—comprising *T. laurifolia* and *E. sessiliflora* (1:1)—demonstrated the most potent antioxidant capacity and effectively triggered early apoptotic responses in LX-2 hepatic stellate cells. Phytochemical analysis identified rosmarinic acid as its major bioactive constituents. In vivo administration of Formula 04 to chlorpyrifos-exposed rats restored acetylcholinesterase activity, reinforced hepatic antioxidant defenses, suppressed lipid peroxidation, normalized erythrocyte indices, and significantly reduced serum AST, with the strongest effects observed at medium and high doses. Histopathological assessment further revealed that high-dose treatment preserved hepatic architecture closely resembling that of normal controls. Importantly, no renal pathology, systemic toxicity, or behavioral abnormalities were observed across the treatment groups. Together, these findings position Formula 04 as a promising hepatoprotective and antifibrotic candidate, with its efficacy likely attributable to synergistic antioxidant and anti-inflammatory mechanisms mediated by rosmarinic acid. Future mechanistic studies should directly compare Formula 04 with its individual components in vivo and evaluate key signaling pathways such as Nrf2/HO-1, NF-κB, and markers of hepatic stellate cell activation (e.g., α-SMA, collagen I/III) to clarify the multi-target pharmacodynamics underlying its protective effects. Moreover, for potential future application in humans, preclinical development should include comprehensive safety assessments in experimental animals following established guidelines such as those of the WHO, OECD, or ICH.

## Figures and Tables

**Table 1:** The proportion of each of the herbs used to develop the detoxification formulas.

No.	Name	Herbs Used (%)			
		*T. coccinea* Vines + Leaves	*M. angustifolia* Roots	*T. laurifolia* Leaves	*E. sessiliflora* Vines
1	TC_01	100	-	-	-
2	MA_01	-	100	-	-
3	TL 01_L	-	-	100	-
4	ES 01_V	-	-	-	100
5	Formula 01	26	54	-	20
6	Formula 02	30	54	16	-
7	Formula 03	50	50	-	-
8	Formula 04	-	-	50	50
9	Formula 05	-	25	50	25
10	Formula 06	-	25	25	50
11	Formula 07	10	30	30	30

**Table 2:** Pharmacognostic Characters of Formula 04.

Specification	Content (% by Dried Weight)
Loss on drying	7.8602 ± 0.04
Total ash	13.3330 ± 0.01
Acid-insoluble ash	4.3062 ± 0.00
Ethanol-soluble extractive value	8.4461 ± 0.04
Water–soluble extractive value	9.6565 ± 0.02

Values are expressed as mean ± S.E.M. (*n* = 3).

**Table 3:** The body weight of rats in the study of anti-pesticide potential of the detoxifying Formula 04.

Experiment Groups	Body Weight (D1)	Body Weight (D9)	Body Weight (D18)
Normal control	255.83 ± 3.75	280.00 ± 8.66	300.83 + 9.26
CPF control	254.17 ± 3.00	264.17 ± 11.29	264.17 ± 5.54 ^a^
Formula 04 low doses	254.17 ± 3.75	273.33 ± 9.46	289.17 ± 12.81
Formula 04 medium doses	256.67 ± 1.67	277.50 + 7.61	278.33 ± 10.14
Formula 04 high doses	256.67 ± 2.47	278.33 ± 5.27	286.67 ± 8.82

Values are expressed as mean ± S.E.M., *n* = 6, ^a^ Significantly different from the normal rat (distilled water), *p* < 0.05.

**Table 4:** Liver and kidney weights of rats in the study of anti-pesticide potential of the detoxifying Formula 04.

Experimental Groups	Liver Weight (g)	Kidney Weight (g)
Normal control	10.82 ± 0.51	1.49 ± 0.07
CPF control	12.34 ± 0.88	1.57 ± 0.08
Formula 04 low doses	11.37 ± 0.73	1.38 ± 0.05
Formula 04 medium doses	11.92 ± 0.64	1.48 ± 0.06
Formula 04 high doses	11.36 ± 0.37	1.37 ± 0.07

Values are expressed as mean ± S.E.M., *n* = 6.

**Table 5:** Effects of detoxifying Formula 04 on hematological values of rats in the study of anti-pesticide potential.

Parameters	Normal Control	CPF Control	Detoxifying Formula 04		
			Low Doses	Medium Doses	High Doses
RBC (×10^6^/µL)	7.88 ± 0.18	8.67 ± 0.14 ^a^	8.43 ± 0.20	8.45 ± 0.15	8.31 ± 0.10
HGB (g/dL)	15.08 ± 0.38	16.00 ± 0.26 ^a^	15.72 ± 0.38	16.08 ± 0.21	15.77 ± 0.23
HCT (%)	45.77 ± 1.16	48.13 ± 0.93	47.82 ± 1.11	48.77 ± 0.69	47.85 ± 0.88
MCV (fL)	58.08 ± 0.80	55.53 ± 0.69 ^a^	56.73 ± 0.64	57.78 ± 0.74 *	57.58 ± 0.91
MCH (pg)	19.15 ± 0.22	18.45 ± 0.25 ^a^	18.63 ± 0.21	19.05 ± 0.23	18.98 ± 0.19
MCHC (g/dL)	32.95 ± 0.12	33.23 ± 0.19	32.87 ± 0.21	32.98 ± 0.17	32.95 ± 0.28
PLT (×10^5^/µL)	8.55 ± 0.24	8.70 ± 0.32	9.08 ± 0.30	8.96 ± 0.16	8.54 ± 0.23
WBC (×10^3^ cells/µL)	5.19 ± 0.49	8.89 ± 1.67 ^a^	5.63 ± 0.52 *	6.20 ± 0.32 *	6.61 ± 0.56
Nu (cells/µL)	0.63 ± 0.06	1.98 ± 0.33 ^a^	0.91 ± 0.15 *	0.83 ± 0.12 *	1.02 ± 0.14 *
Lymph (cells/µL)	4.24 ± 0.41	6.37 ± 1.35 ^a^	4.19 ± 0.51 *	5.03 ± 0.27	4.98 ± 0.38
Mono (cells/µL)	0.27 ± 0.06	0.46 ± 0.09	0.47 ± 0.09	0.29 ± 0.03	0.53 ± 0.19
E (cells/µL)	0.06 ± 0.01	0.07 ± 0.02	0.05 ± 0.01	0.06 ± 0.01	0.09 ± 0.03
Ba (cells/µL)	0.00 ± 0.00	0.01 ± 0.01 ^a^	0.00 ± 0.00 *	0.00 ± 0.00 *	0.00 ± 0.00 *

Values are expressed as mean ± S.E.M., (*n* = 6), ^a^ Significantly different from the normal rats (distilled water), *p* < 0.05, * Significantly different from the control group (distilled water + chlorpyrifos), *p* < 0.05.

**Table 6:** Effects of the detoxifying Formula 04 on blood chemistry values of rats in the study of the anti-pesticide potential.

Parameters	Normal Control	CPF Control	Detoxifying Formula 04		
			Low Doses	Medium Doses	High Doses
BUN (mg/dL)	15.18 ± 1.33	18.25 ± 2.05	17.08 ± 2.71	12.68 ± 0.59 *	13.38 ± 0.52 *
Creatinine (mg/dL)	0.59 ± 0.02	0.62 ± 0.02	0.60 ± 0.02	0.58 ± 0.01	0.60 ± 0.02
Total protein (g/dL)	5.12 ± 0.11	5.37 ± 0.18	5.25 ± 0.10	5.37 ± 0.09	5.38 ± 0.06 *
Albumin (g/dL)	2.90 ± 0.05	2.95 ± 0.09	2.97 ± 0.08	3.30 ± 0.36	3.00 ± 0.03
Total bilirubin (mg/dL)	0.12 ± 0.01	0.13 ± 0.01	0.15 ± 0.02	0.12 ± 0.01	0.15 ± 0.01
Direct bilirubin (mg/dL)	0.07 ± 0.00	0.07 ± 0.00	0.08 ± 0.02	0.08 ± 0.01	0.09 ± 0.00
AST (U/L)	95.67 ± 8.91	206.67 ± 31.02 ^a^	132.00 ± 9.56 *	99.00 ± 9.71 *	132.50 ± 19.08 *
ALT (U/L)	22.17 ± 1.99	33.33 ± 3.95	33.00 ± 5.36	29.00 ± 5.88	48.83 ± 13.88
ALP (U/L)	121.17 ± 9.01	91.67 ± 9.91	112.00 ± 8.85	109.00 ± 14.92	120.50 ± 12.60

Values are expressed as mean ± S.E.M. (*n* = 6), ^a^ Significantly different from the normal rats (distilled water), *p* < 0.05, * Significantly different from the control group (distilled water + chlorpyrifos), *p* < 0.05.

## Data Availability

The data presented in this study are available in this article.
